# Dietary Resveratrol Does Not Affect Life Span, Body Composition, Stress Response, and Longevity-Related Gene Expression in *Drosophila melanogaster*

**DOI:** 10.3390/ijms19010223

**Published:** 2018-01-11

**Authors:** Stefanie Staats, Anika E. Wagner, Bianca Kowalewski, Florian T. Rieck, Sebastian T. Soukup, Sabine E. Kulling, Gerald Rimbach

**Affiliations:** 1 Institute of Human Nutrition and Food Science, University of Kiel, Hermann-Rodewald-Strasse 6, D-24118 Kiel, Germany; b-i-b-i@o2online.de (B.K.); rimbach@foodsci.uni-kiel.de (G.R.); 2 Institute of Nutritional Medicine, University of Lübeck, Ratzeburger Allee 160, D-23538 Lübeck, Germany; Anika.Wagner@uksh.de; 3 Department of Safety and Quality of Fruit and Vegetables, Max Rubner Institute, Haid-und-Neu-Strasse 9, D-76131 Karlsruhe, Germany; florian@rieck.ru (F.T.R.); sebastian.soukup@mri.bund.de (S.T.S.); sabine.kulling@mri.bund.de (S.E.K.)

**Keywords:** resveratrol, *Drosophila*, healthy ageing, life span, longevity

## Abstract

In this study, we tested the effect of the stilbene resveratrol on life span, body composition, locomotor activity, stress response, and the expression of genes encoding proteins centrally involved in ageing pathways in the model organism *Drosophila melanogaster*. Male and female *w^1118^ D. melanogaster* were fed diets based on sucrose, corn meal, and yeast. Flies either received a control diet or a diet supplemented with 500 µmol/L resveratrol. Dietary resveratrol did not affect mean, median, and maximal life span of male and female flies. Furthermore, body composition remained largely unchanged following the resveratrol supplementation. Locomotor activity, as determined by the climbing index, was not significantly different between control and resveratrol-supplemented flies. Resveratrol-fed flies did not exhibit an improved stress response towards hydrogen peroxide as compared to controls. Resveratrol did not change mRNA steady levels of antioxidant (*catalase*, *glutathione-S-transferase*, *NADH dehydrogenase*, *glutathione peroxidase*, *superoxide dismutase 2*) and longevity-related genes, including *sirtuin 2*, *spargel*, and *I’m Not Dead Yet*. Collectively, present data suggest that resveratrol does not affect life span, body composition, locomotor activity, stress response, and longevity-associated gene expression in *w^1118^ D. melanogaster*.

## 1. Introduction

Diet is an important determinant of health and disease prevention. Epidemiological data on the consumption of foods rich in fruits and vegetables suggests that secondary plant metabolites may favour health and successful ageing [1].

The traditional Asian and the Mediterranean diets are rich in fruits and vegetables [2]. There are specific plant bioactives, which predominantly occur in the Mediterranean (e.g., resveratrol from red wine, hydroxytyrosol from olives) and in the Asian diets (e.g., isoflavones from soybean and epigallocatechin gallate from green tea). In this context, we have recently introduced the concept of the so-called “MediterrAsian” diet combining foods of the traditional Asian as well as Mediterranean diet as a promising dietary strategy in chronic disease prevention [2].

*Drosophila melanogaster* is widely used as a model organism in ageing studies. *Drosophila* exhibits a relatively short life span of 60 to 90 days, which makes it particularly attractive for life span studies [3,4]. Furthermore, in recent years, the fruit fly has also been increasingly recognised as a model organism in nutrition research. Feed intake, body composition, locomotor activity, gut function, composition of the microbiota, ageing, as well as life span can be systematically determined in *Drosophila* in response to dietary factors [5,6,7,8,9,10,11]. Moreover, diet-induced pathophysiological mechanisms including both intestinal and systemic inflammatory processes [12,13,14,15,16,17], and stress response against various triggers like reactive oxygen species, alcohol, acids, or heat [6,12,18,19] may be evaluated in the fruit fly under defined experimental conditions. We have recently shown that secondary plant metabolites including isoflavones [5], green tea catechins [10], and isothiocyanates [20] are capable of improving health status and survival in male *D. melanogaster*.

The stilbene *trans*-resveratrol (3,4′,5-trihydroxystilbene) has been widely suggested as a putative “anti-ageing” molecule, e.g., in *Saccharomyces cerevisiae* [21,22,23], *Caenorhabditis elegans* [24,25,26], and killifish [27,28,29]. However, literature is contradictory regarding the life span modulating properties of resveratrol in *D. melanogaster* [24,30,31,32]. Furthermore, resveratrol mostly failed to improve life span in studies conducted in mice [33]. Several mechanisms, including induction of autophagy and sirtuins [34,35,36,37,38,39], modulation of IGF signalling [26,40,41], improvement of stress response [42,43,44,45,46], endogenous antioxidant defence [43,47,48], mitochondrial function [41,49,50,51], as well as anti-inflammatory properties [52,53,54,55,56,57,58,59], have been suggested by which resveratrol may counteract the ageing process. Moreover, there is literature data indicating that resveratrol may affect body weight [60,61,62,63,64,65], body composition [62,64,66], and metabolism [65,66,67,68] in different species—however, data are partly contradicting.

Although resveratrol has been shown to increase the life span in short-lived species like worms (*C. elegans*) [24,25] and killifish [27,28], the role of resveratrol in the fruit fly is less clear. Therefore, the aim of the present study was to systematically investigate the effect of dietary resveratrol on life span, body composition, stress response, and longevity-associated gene expression in *D. melanogaster*.

## 2. Results

**Figure 1:**
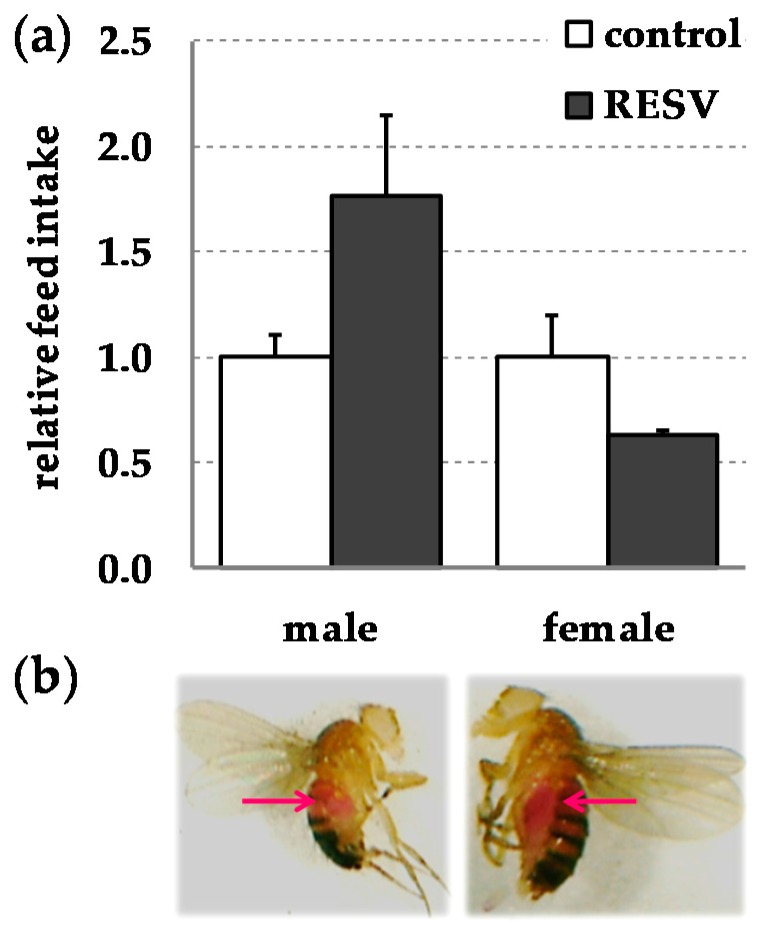
Dietary resveratrol (RESV; 500 µmol/L) does not affect feed intake in *w^1118^ D. melanogaster*. (**a**) Relative feed intake in male and female flies following a five-day feeding period with a RESV-supplemented or a control diet. Flies were administered the experimental diets for five days prior to administration of a sulforhodamine B (0.2% *w*/*v*)-supplemented medium for 8 h. Feed intake was quantified via fluorometric measurements. Feed intake of RESV-treated males and females was normalised to the feed intake of their control fed counterparts, respectively. Bars show means ± SEM comprising 60–100 flies/group. Statistics: 2-sided Student’s *t*-test and Mann–Whitney U.; (**b**) Bright-field pictures of male and female *w^1118^* flies administered with sulforhodamine B for 8 h. Arrows point to pink-coloured body parts due to the sulforhodamine B ingestion.

Since feed intake may affect body composition, metabolism, locomotor activity, and life span, we monitored the feed intake of *D. melanogaster* in the presence and absence of resveratrol by the food-dye-based “sulforhodamine B gustatory assay” [5,69]. Under the conditions investigated there were no significant differences in feed intake between resveratrol-supplemented flies and controls both in males (*p* = 0.162) and females (*p* = 0.126) (Figure 1).

Accordingly, resveratrol-supplemented and control-fed flies exhibited similar fat, protein, and glucose contents (Table 1), whereby flies showed a rather heterogeneous response to the dietary resveratrol treatment as revealed by higher standard errors. Solely the protein content was slightly increased in resveratrol-fed males compared to controls. Thus, overall body composition of *D. melanogaster* remained largely unchanged in response to dietary resveratrol supplementation.

**Figure 2:**
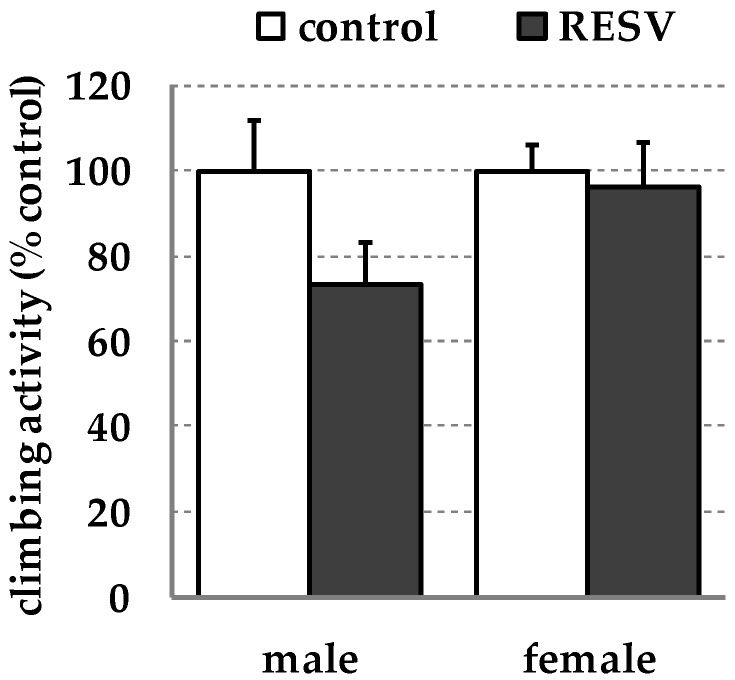
Dietary resveratrol (RESV; 500 µmol/L) does not affect locomotor activity in *w^1118^ D. melanogaster*. Relative climbing activity of male and female flies following a thirty-day feeding period in the presence or absence of resveratrol. Locomotor activity was quantified via the rapid iterative negative geotaxis (RING) assay. Bars show means ± SEM from 20 measurements/group derived from two independent experiments. Statistics: Mann–Whitney U. RESV: resveratrol (500 µmol/L).

Locomotor activity of *D. melanogaster* was determined by calculating the climbing score applying the so-called RING assay [70,71]. Under the conditions investigated, locomotor activity was similar between control and resveratrol fed flies both in males (*p* = 0.092) and females (*p* = 0.743) as shown in Figure 2.

**Figure 3:**
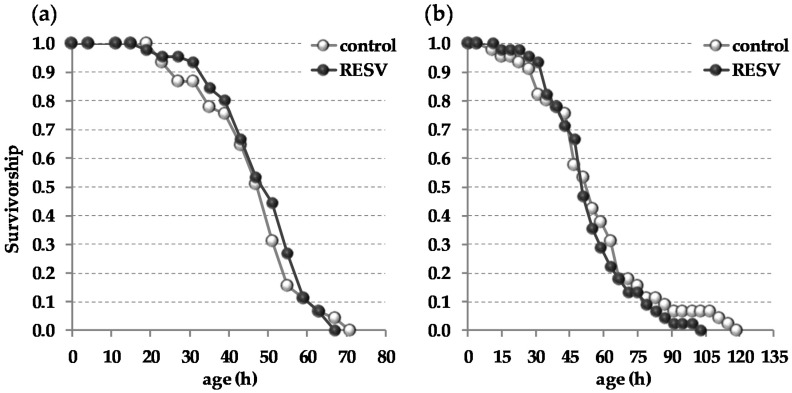
Dietary resveratrol (RESV; 500 µmol/L) does not improve stress resistance of *w^1118^ D. melanogaster* against reactive oxygen species. Flies received a resveratrol-supplemented or a control diet for ten days prior to the exposure to hydrogen peroxide (10% *w*/*v* diluted in a 5% *w*/*v* sucrose solution). Dead flies were steadily counted every four hours. (**a**) Survival curve of male and (**b**) female flies. The stress resistance experiment was independently performed three times with 45 flies/group each revealing similar results. Statistics: Log-Rank.

The hydrogen peroxide-based stress resistance assay is well established and suitable to examine both direct and indirect antioxidant effects of secondary plant metabolites in fruit flies [6,72,73,74,75]. In order to test flies for stress resistance against reactive oxygen species, male and female *w^1118^* were challenged with hydrogen peroxide (10% *w*/*v* diluted in a 5% *w*/*v* sucrose solution) following a ten-day feeding period with a resveratrol-supplemented or a control diet. The hydrogen peroxide administration dramatically increased mortality of both male and female *D. melanogaster* as reported in the literature. However, there was no significant advantage for survival when flies received dietary resveratrol prior to hydrogen peroxide challenge as compared to controls (Figure 3). Both male and female flies did not benefit from dietary resveratrol supplementation or even displayed slightly reduced mean and median survival rates compared to their control-fed counterparts.

Accordingly, mRNA expression levels of genes encoding antioxidant enzymes were not significantly modulated by resveratrol ingestion (Table 2).

Several studies suggest that resveratrol may affect the life span of model organisms. Therefore, we determined mean, median, and maximum life span of flies in response to the resveratrol treatment. However, dietary resveratrol did not change mean, median, and maximum life span of male and female *w^1118^ D. melanogaster* in general (Table 3). Resveratrol ingestion rather decreased than increased mean, medium, and maximum life span in both males and females compared to the respective control groups in the majority of the assays performed within this study (Table 3), although resveratrol-dependent differences in life span remained mostly non-significant.

Furthermore, we monitored mRNA steady levels of various genes encoding proteins that have been reported to be related to ageing and longevity in the fruit fly. Thus, mRNA levels of the health and life span associated genes *I’m Not Dead Yet* (*INDY*) [76], *sirtuin* (*Sir2*) [77], and *spargel* (*srl*) [78] were determined by quantitative real time PCR in whole body homogenates. Similar to our life span data, none of these genes were significantly regulated on the transcript level following dietary resveratrol supplementation. Relative transcript levels are summarized in Table 4.

Resveratrol displays a rather low bioavailability; hence tissue distribution may affect its efficacy to modulate metabolism and life span [79]. Therefore, we determined resveratrol concentrations in the whole body homogenates of male and female flies. We recovered substantial quantities of resveratrol in the whole body homogenates of resveratrol-treated flies, whereas no resveratrol was detected in homogenates of controls (limit of detection 0.49 nmol/g flies). These data suggest that resveratrol was readily taken up from the diet. Interestingly, female flies fed the resveratrol-supplemented diet exhibited a 2.2-fold higher resveratrol body concentration as compared to male flies (20.98 ± 0.44 vs. 9.60 ± 2.80 nmol/g fly; means ± SD; N = 54 flies/group following a ten-day feeding period).

## 3. Discussion

Several studies in lemurs, mice, honey bees, and humans suggest that resveratrol may affect food intake due to its bitter taste [65,80,81,82,83], which may in turn affect stress response and metabolism. However, in the current fly study, we did not observe significant differences in feed intake between groups (Figure 1). Although literature data suggest that resveratrol may affect body weight and body composition [60,61,62,63,64,65,66,84], we did not find any significant changes in these parameters, except for the slight increase in protein content in males, following dietary resveratrol supplementation (Table 1).

Furthermore, we did not observe changes in both locomotor activity and life span in resveratrol-fed flies as compared to controls (Figure 2; Table 3). One limitation of our present study is that only one dietary resveratrol concentration has been investigated and no dose-response analysis has been performed. Thus, we cannot fully exclude the possibility that higher or lower dietary resveratrol concentrations may modulate overall health status and life span in *D. melanogaster* as indicated by other but rather contradictory studies [24,30,31,85,86]. Therefore, further studies may include additional resveratrol concentrations and treatment periods for body weight and composition measurements, transcript analyses, and oxidative stress resistance evaluation. Due to comparable high variances in the treatment groups, the inclusion of further fly strains should be also considered.

We have recently conducted a systematic literature review concerning the effect of resveratrol on life span in various model organisms. Interestingly, resveratrol supplementation has been reported to increase life span in approximately 60% of the studies conducted in model organisms [33]. However, literature data is rather inconsistent, suggesting that the life span effects of resveratrol vary in relation to the model organism. Furthermore, it should be considered that other factors such as dose, gender, genetic background, and diet composition [35,87] may contribute to the high variance in the life span prolonging ability of resveratrol in the fruit fly.

Several plant bioactives, including lutein, epicatechin, epigallocatechin gallate, and apple polyphenols, have been reported to counteract reactive oxygen species induced mortality in *D. melanogaster* [6,88,89]. It is suggested that the protective action of these plant bioactives may be partly mediated via the induction of the transcription factor cnc, which represents an ortholog of mammalian Nrf2 in the fruit fly [90], and its target genes exhibiting antioxidant activity [89,91,92]. However, under the conditions investigated resveratrol did not improve the survival of flies treated with hydrogen peroxide as compared to controls (Figure 3). Accordingly, the relative transcript levels of cnc target genes and further antioxidant enzymes such as *glutathione-S-transferase*, *glutathione peroxidase*, *NADH dehydrogenase*, *superoxide dismutase*, and *catalase* remained largely unchanged in response to the resveratrol treatment (Table 2).

Cell culture as well as in vivo studies suggest that resveratrol may modulate the expression of genes encoding proteins that are centrally involved in ageing related pathways and suppresses DNA damage, thereby affecting the ageing process [93,94,95,96]. Among others, sirtuins seem to be important molecular targets of resveratrol [49,97,98]. Sir2 is an important modulator of the ageing process and locomotor activity in the fruit fly [77,86,99]. Moderate induction of Sir2 expression is associated with life span elongation in *D. melanogaster* in response to both nutrient composition as well as dietary plant bioactives [5,77,99,100,101,102]. In contrast, mutant fly strains with diminished or absent Sir2 expression display a markedly shortened life span and lack responsiveness to life span extending dietary interventions [31,99,101]. However, in the present study, *Sir2* mRNA levels were not affected in whole body homogenates of *D. melanogaster*. The same holds true for other genes previously reported to affect ageing and life span in the fruit fly, including *INDY* and *srl* (Table 4), being in accordance with the ineffectiveness of resveratrol to prolong life span in *w^1118^* males and females (Table 3). It should be considered that *INDY* expression is not inevitably associated with longevity in the *w^1118^ D. melanogaster* strain [103]. Moreover, it needs to be taken into account that, in the present study, gene expression was determined in whole body homogenates and not in specific tissues and organs of *D. melanogaster*. Thus, it could be possible that resveratrol may have affected gene expression in distinct tissues of the fly, which was however beyond the scope of the current study.

Unlike in the present study in *w^1118^ D. melanogaster*, resveratrol was shown to increase life span in the short-lived killifish [27,28] and in laboratory mice in dependence of their genetic background [41,104]. Besides differences in the dietary resveratrol concentrations and durations of the experimental trials, there may be also species-specific differences in resveratrol metabolism [33].

Importantly, resveratrol whole body concentrations in both male and female flies were elevated in response to dietary resveratrol supplementation. Interestingly, female flies exhibited a whole body resveratrol concentration more than twice as high as male flies, which warrants further investigations into the underlying mechanisms. Studies by Wang et al. and Zou et al. [32,87] suggested that female but not male *D. melanogaster* may benefit from dietary resveratrol supplementation in terms of life span prolongation in dependence of the macronutrient composition of the diet. This may be partly related to a higher resveratrol body concentration in female vs. male flies as observed in the present study. The storage of fat diverges in male and female fruit flies starting in the early adulthood while the extent of the sex-specific deviation depends on the fly strain [105,106]. Thus, difference in whole body resveratrol content between male and female flies may also partly depend on the higher fat content in *w^1118^* females compared to males (triglyceride level in ten-day-old males: 13.2 ± 0.7 ng/µg fly; females: 32.7 ± 2.8 ng/µg fly; *p* < 0.001) resulting in an increased accumulation of the primarily fat-soluble resveratrol.

In conclusion, present data suggest that dietary resveratrol, although sufficiently absorbed, does not affect life span, body composition, stress response, and longevity-related gene expression in male and female *w^1118^ D. melanogaster*.

## 4. Materials and Methods

### 4.1. Fly Strain and Husbandry

The *Drosophila melanogaster* strain *w^1118^* (Bloomington Drosophila Stock Center #5905, Indiana University, Bloomington, IN, USA) was used for all experiments. *w^1118^* flies were maintained on a standard diet at 25 °C and 60% humidity with a 12/12 h light–dark cycle. Standard medium was prepared as described previously [5]. The experimental diet consisted of sucrose (5% *w*/*v*; Carl Roth, Karlsruhe, Germany), Agar Type II (0.5% *w*/*v*; Dutscher Scientific, Grays, UK), corn meal (8.6% *w/v*; Dutscher Scientific), and inactive dry yeast (5% *w*/*v*; Dutscher Scientific), while Tegosept (0.3% *w*/*v*; Dutscher Scientific) and propionic acid (0.3% *v*/*v*; Carl Roth) served as preservatives. Resveratrol was purchased from Carl Roth and was dissolved in dimethyl sulfoxide (DMSO; Carl Roth) at a concentration of 100 mmol/L. This resveratrol stock solution was stored at −80 °C until supplementation of the experimental diet at a final concentration of 500 µmol/L. This concentration was chosen based on contradictory literature data reporting both beneficial [30,35,85] and detrimental [24,32] effects of oral resveratrol administration on the fruit fly that were observed in a concentration range between 100 and 1000 µmol/L. To ensure sufficient supply and absorption of resveratrol, 500 µmol/L were orally applied in this study. DMSO (0.5% *v*/*v*) served as the vehicle control. For all experiments, flies received either a resveratrol-supplemented (500 µmol/L) or a control diet and were reared on the experimental diets at a population density of 25 flies/vial.

### 4.2. Life Span Analyses

Newly enclosed synchronized flies were permitted to mate for two days as described previously [5]. Sexual activity may act as a confounding variable in life span experiments [107,108] as frequent mating shortens life expectancy in *D. melanogaster* [107,109]. Therefore, flies were separated according to sex two days past enclosure and were housed in single-sex cohorts. The flies were fed the resveratrol-supplemented or the control diet life-long while transferred to fresh medium three times a week. The number of dead flies was recorded at each transfer until all flies were dead. The experiment was independently performed three times with 150 flies/group, respectively.

### 4.3. Gustatory Assay

Sulforhodamine B (0.2% *w*/*v*; Sigma-Aldrich Chemie GmbH, Taufkirchen, Germany) was used to determine food intake in both male and female flies fed a resveratrol-supplemented or a control diet. Flies were reared on the experimental diet for five days prior performing the food-dye-based gustatory assay according to the protocol described elsewhere [5,69]. This feeding period was chosen as food consumption in older flies remarkably declines in comparison to early-life feeding levels [110]. The gustatory assay was independently performed two times.

### 4.4. Body Weight and Body Composition

Flies received a resveratrol-supplemented or a control diet for ten days. Body weight was estimated as described in [5], the same applies to the quantification of whole body triglyceride, protein and glucose levels. Average body weight of male and female *w^1118^* was calculated from two independent experiments with 51–137 flies/group, respectively (178–224 flies/group in total). Body composition data were collected from three independent experiments comprising 5–15 flies/group, respectively (20–35 flies/group in total).

### 4.5. Locomotor Activity

Locomotor activity can be easily evaluated in *D. melanogaster* by applying the rapid iterative negative geotaxis (RING) assay [70,71] indicating the health status of fruit flies. Climbing speed was determined in both male and female flies by performing the RING assay as previously described in detail in [5] following oral administration of a resveratrol-supplemented or a control diet for thirty days. As locomotor activity declines with age [11] and senescence is functionally associated with the flies’ climbing ability reflecting the functional status of muscle and locomotor function [111], flies were pre-treated for thirty days to investigate a putative anti-ageing effect of resveratrol in *D. melanogaster*. The experiment was performed two times comprising ten repetitive measurements each (N = 100).

### 4.6. Oxidative Stress Resistance

Resistance against reactive oxygen species was investigated by applying the hydrogen peroxide stress test as described previously [12]. As H_2_O_2_ generates hydroxyl radicals in the presence of metal ions [6], it was used to assess the resistance of male and female *w^1118^* flies that were pre-fed with a resveratrol (500 µmol/L)-supplemented or a control diet for ten days. This pre-feeding period was chosen as flies exhibited adequate whole body resveratrol concentrations that allowed the investigation of putative resveratrol-dependent antioxidant effects in vivo. The experiment was independently performed three times including 45 flies/group, respectively.

### 4.7. RNA Isolation and qRT-PCR

Flies were orally administered a resveratrol (500 µmol/L)-supplemented or a control diet for ten days. Whole flies (ten per sample) were homogenised in a TissueLyser II (Qiagen, Hilden, Germany) prior to total RNA isolation with the help of TriFast reagent (peqlab, Erlangen, Germany) according to the manufacturer’s protocol. RNA concentration was determined via NanoDrop measurements (NanoDrop2000c; ThermoScientific, Waltham, MA, USA). mRNA expression was quantified by qRT-PCR measurements using the SensiFast SYBR No-ROX One-Step Kit (Bioline, London, UK) and a Rotor-Gene 6000 real-time PCR cycler (Corbett/Qiagen). Relative mRNA concentrations were calculated using a standard curve. The expression of the longevity associated genes (*INDY*, F: GATTGGTTGTGTTCCTGGTG, R: CGTCACATAGAGAGGCAAGG; *Sir2*, F: CCGTTACTGAGGAGGAGCTG, R: GTAGATCGCACACGTCCTTG; *srl*, F: CTCTTGGAGTCCGAGATCCGCAA, R: GGGACCGCGAGCTGATGGTT [78]), and of the antioxidant enzymes (*Cat*, F: CCTCTGATTCCTGTGGGCAA, R: GACGACCATGCAGCATCTTG; *GstD2*, F: GTCTACTTCGCAGGCATCAC, R: CTTCTCGATCCAGGCCTTGA; *ND-75*, F: CGGACATTAACTACACGGGC, R: CAATCTCGGAGGCGAAACG; *PHGPx*, F: CCTCAACTTCCCGTGCAATC, R: CCATTCACATCGACCTTGGC; *Sod2*, F: AACGCAGATATGTTCGTGGC, R: GGTGATGCAGCTCCATGATC) was normalized to the expression of the housekeeping genes *α-Tubulin at 84B* (*αTub84B*) and *Ribosomal protein L32* (*RpL32*), respectively. Experiment was independently performed two times with 20 flies/group, respectively.

### 4.8. Whole Body Resveratrol Concentration

Newly hatched two-day-old flies were orally administered a resveratrol (500 µmol/L)-supplemented or a control diet for ten days. Whole flies were stored at −80 °C until analysis. For analysis, whole flies (27 flies/sample) were mixed with 200 µL ice-cold aqueous triethylammonium acetate solution (1 mol/L, pH = 7.0) followed by adding 1 mL of an ice-cold acetonitril–methanol mixture (1:1, *v*/*v*). Afterwards, 1-mm silica spheres were added and flies were treated with a FastPrep homogenizer (3 × 15 s, 4 m/s) with intermediate cooling periods on ice (FastPrep-24, MP Biomedicals, Solon, OH, USA). Homogenates were transferred to a new sample tube. The residual silica spheres were washed with 100 µL of an ice-cold acetonitrile–methanol mixture (1:1, *v*/*v*) and this washing fraction was combined with the transferred homogenates. Next, homogenates were first stirred on a vortex mixer for 10 s and then treated in an ultrasonic bath for 10 min. Once again, homogenates were stirred on a vortex mixer for 10 s followed by a centrifugation of the samples (23,100× *g*; 10 min; 0 °C). Supernatants were transferred to new sample tubes und evaporated to dryness using a SpeedVac (SPD131; Thermo Electron LED GmbH, Langenselbold, Germany). Residues were first dissolved in 70 µL of 30% (*v*/*v*) methanol in water and then centrifuged at 23,100× *g* for 10 min. Supernatants were transferred to a HPLC vial and analysed by LC-MS. A TripleTOF 5600 mass spectrometer (AB Sciex, Darmstadt, Germany) equipped with a 1290 Infinity LC system (Agilent, Waldbronn, Germany) was used. The LC-MS system was controlled by the software Analyst TF 1.6.0. LC separation was carried out on an Agilent Eclipse Plus C18 column (150 mm × 3.0 mm internal diameter, 3.5 µm; Agilent). Eluent A was an aqueous ammonium formiate buffer (25 mmol/L, pH = 3.0) and eluent B was an acetonitrile–methanol mixture (1:1, *v*/*v*). A linear gradient was used with a flow rate of 0.6 mL/min and the following elution profile: 0–1 min, isocratic with 10% B; 1–19 min, from 10 to 28% B; 19–33 min from 28 to 70% B; 33–34 min, from 70 to 95% B; 34–39 min, isocratic with 95% B; 39–40 min, from 95 to 10% B; and 40–45 min, isocratic with initial conditions. The column oven was set to 40 °C and the injection volume was 20 µL. The DuoSpray source was operated in negative ESI mode using the following source parameters: curtain gas, 35 psi; ion spray voltage, −4500 V; ion source gas 1, 60 psi; ion source gas 2, 70 psi; ion source gas 2 temperature, 650 °C. The MS full scans were recorded from *m*/*z* 100 to 800 with an accumulation time of 200 ms, a declustering potential of −110 V and a collision energy voltage of −10 V. The MS/MS spectra (product ion) were recorded from *m*/*z* 50 to 600 in the high-sensitivity mode with an accumulation time of 80 ms, a collision energy voltage of −45 V, and a collision energy spread of 25 V. Nitrogen was used as collision gas. Analysis of data was performed with the MultiQuant 2.1.1 and PeakView 1.2.0.3 software (AB Sciex, Darmstadt, Germany). *trans*-Resveratrol was identified by retention time and MS/MS spectrum. Accurate XICs (10 mDa extraction width) were used to monitor and quantify the analytes. In detail, *trans*-resveratrol was quantified by an external calibration using commercial reference compound (Sigma-Aldrich, Deideshofen, Germany). Therefore, control flies were spiked with 5 µL of the specific standard solution (concentration range between 0.8 and 100 µmol/L *trans*-resveratrol in DMSO) and these spiked samples were worked up as described above. A best fit line was obtained by linear regression using a weighting of 1/x. Whole body resveratrol measurements comprised 54 flies/group, respectively.

### 4.9. Statistics

Life span and survival rates following H_2_O_2_ treatment were analysed using the DLife software (Winchecker version 3.0 [112]). Values are given as means and were statistically compared via a Log-Rank Test based on R (i386 version 3.1.0). All other data are given as means ± SEM, except otherwise mentioned, and were statistically analysed by applying SPSS (version 24; SPSS Inc., Munich, Germany). Data were tested for normality of distribution (Kolmogorov–Smirnov and Shapiro–Wilk) and mean comparisons were carried out using a 2-sided Student’s *t*-test. If the assumption of a normal distribution was violated, the non-parametric Mann–Whitney U. test was used. Significance was accepted at *p*-values < 0.05.

## Figures and Tables

**Table 1:** Changes in body weight and body composition of male and female *w^1118^ D. melanogaster* in dependence of dietary resveratrol (RESV; 500 µmol/L) administration for ten days compared to controls.

Parameter	Male			Female		
	Control	RESV ^1^	*p*-Value	Control	RESV ^1^	*p*-Value
Body weight (µg/fly)	778 ± 46	774 ± 42	0.953	1222 ± 43	1223 ± 77	0.989
Triglycerides (% control)	100 ± 9.0	165 ± 31	0.083	100 ± 5.1	95.5 ± 13	0.750
Protein (% control)	100 ± 1.8	121 ± 7.5	**0.035**	100 ± 2.0	103 ± 5.3	0.679
Glucose (% control)	100 ± 12	115 ± 21	0.500	100 ± 14	156 ± 34	0.100

All data are shown as means ± SEM from three independent experiments comprising 178–224 flies/group (body weight) and 20–35 flies/group (body composition). Outliers were removed. Statistics: 2-sided Student’s *t*-test and Mann–Whitney U. ^1^ RESV: resveratrol (500 µmol/L).

**Table 2:** Relative mRNA expression of antioxidant enzymes in female *w^1118^ D. melanogaster* in dependence of resveratrol (RESV; 500 µmol/L) administration for ten days compared to controls.

Target	mRNA Expression Level vs. Control	*p*-Value ^6^
*Cat* ^1^	0.87 ± 0.15	0.700
*GstD2* ^2^	1.02 ± 0.22	0.948
*ND-75* ^3^	0.99 ± 0.22	0.963
*PHGPx* ^4^	0.89 ± 0.17	0.741
*Sod2* ^5^	1.03 ± 0.18	0.930

Data are shown as means ± SEM comprising 40 flies/group from two independent experiments; *α-Tubulin at 84B* (*αTub84B*) and *Ribosomal protein L32* (*RpL32*) served as the housekeeping genes. Statistics: 2-sided Student’s *t*-test and Mann–Whitney U. ^1^ *Cat*: *Catalase*; ^2^ *GstD2*: *Glutathione S transferase D2*; ^3^ *ND-75*: *NADH dehydrogenase* (ubiquinone) 75 kDa subunit; ^4^ *PHGPx*: *PHGPx* with glutathione peroxidase activity; ^5^ *Sod2*: *Superoxide dismutase 2* (Mn). ^6^ *p*-value indicates differences in relative mRNA expression in whole body RNA extracts of resveratrol treated flies compared to controls.

**Table 3:** Differences in mean, median, and maximum survival rates (%) of male and female *w^1118^ D. melanogaster* in dependence of life-long dietary resveratrol (RESV; 500 µmol/L) administration compared to controls.

Trial	Males				Females			
	Mean	Median	Max. ^1^	*p*-Value ^2^	Mean	Median	Max. ^1^	*p*-Value ^2^
No. 1	+15.2	+2.50	+1.03	0.307	−8.20	−7.50	−14.1	0.066
No. 2	−11.8	−19.6	+3.04	0.345	−14.7	−46.2	−1.04	0.099
No. 3	−33.1	−56.3	−9.35	<0.001	+15.9	±0.00	+1.06	0.341

The life span experiment was independently performed three times (Trial No. 1–3) comprising 150 flies/group in each experiment. Changes in mean, medium, and maximum life span (% control) of RESV treated males and females in comparison to the respective control group are shown for each single experiment. RESV did not improve the life span of male and female *w^1118^ D. melanogaster*. Statistics: Log-Rank. RESV: resveratrol (500 µmol/L); data are shown as means. ^1^ max.: maximum lifespan defined as the mean survival of the longest-lived 10%; ^2^ *p*-value indicates differences in overall survival benefit of resveratrol treated flies compared to controls.

**Table 4:** Relative mRNA expression of longevity-associated genes in male and female *w^1118^ D. melanogaster* in dependence of resveratrol (RESV; 500 µmol/L) administration for ten days compared to controls.

Sex	Transcript					
	*Sir2* ^1^	*p*-Value ^4^	*INDY* ^2^	*p*-Value ^4^	*Srl* ^3^	*p*-Value ^4^
Male	0.90 ± 0.19	0.700	1.13 ± 0.15	0.437	1.77 ± 0.74	0.375
Female	0.83 ± 0.12	0.405	1.02 ± 0.14	0.913	1.12 ± 0.19	0.593

Data are shown as means ± SEM comprising 40 flies/group in total from two independent experiments; *α-Tubulin at 84B* (*αTub84B*) and *Ribosomal protein L32* (*RpL32*) served as the housekeeping genes. Statistics: 2-sided Student’s *t*-test and Mann–Whitney U. ^1^ *Sir2*: *Sirtuin 2*; ^2^ *INDY*: *I’m Not Dead Yet*; ^3^ *srl*: *spargel*; ^4^ *p*-value indicates differences in relative mRNA expression in whole body RNA extracts of resveratrol-treated flies compared to controls.

## Abbreviations

*Cat*	Catalase
*GstD2*	Glutathione S transferase D2
*INDY*	I’m Not Dead Yet
Max.	maximum
*ND-75*	NADH dehydrogenase (ubiquinone) 75 kDa subunit
*PHGPx*	PHGPx with glutathione peroxidase activity
RESV	resveratrol
*Sir2*	Sirtuin 2
*Sod2*	Superoxide dismutase 2 (Mn)
*srl*	spargel
